# Cost-effectiveness analysis of toripalimab combined with nab-paclitaxel as a first-line treatment for advanced TNBC in the US

**DOI:** 10.1371/journal.pone.0320727

**Published:** 2025-04-01

**Authors:** Ping Chen, Dan Qiao, Liping Xiao, Guiya Deng, Qing Yang, Rendi Tian

**Affiliations:** 1 Department of Nursing, Sichuan Clinical Research Center for Cancer, Sichuan Cancer Hospital & Institute, Sichuan Cancer Center, University of Electronic Science and Technology of China, Chengdu, China,; 2 Department of Head and Neck Surgery, Sichuan Clinical Research Center for Cancer, Sichuan Cancer Hospital & Institute, Sichuan Cancer Center, University of Electronic Science and Technology of China, Chengdu, China; Fujian Provincial Hospital, China

## Abstract

**Introduction:**

Initial immunotherapy outcomes with toripalimab suggest a potential paradigm shift in the treatment of advanced triple-negative breast cancer (TNBC), promising extended survival for patients. However, its cost-effectiveness in the treatment of TNBC within the US health care context remains to be determined.

**Methods:**

A 5-year Markov model was developed using data from the TORCHLIGHT study to evaluate the cost-effectiveness of toripalimab plus nab-paclitaxel as a first-line therapy for metastatic or recurrent TNBC in the US. The model incorporated efficacy and safety data, literature-derived costs and utilities, and calculated ICERs. Sensitivity analyses were conducted to assess the impact of variable uncertainties on the outcomes.

**Results:**

Toripalimab combined with nab-P chemotherapy for TNBC patients resulted in an additional 2.68 life years (LYs) and 1.72 quality-adjusted life years (QALYs), with an ICER of $593,750 per QALY. Sensitivity analyses indicated that the cost and survival utility of toripalimab significantly influence patient outcomes. At a $100,000/QALY WTP threshold, combination therapy was not cost-effective compared with nab-P alone.

**Conclusions:**

Our analysis suggests that, from a US health care system perspective, toripalimab in combination with chemotherapy does not demonstrate a significant cost-effective advantage over nab-P chemotherapy as a first-line treatment for patients with TNBC at a WTP threshold of $100,000/QALY and has a limited impact on US health care policy and clinical practice.

## Introduction

Globally, breast cancer is the most prevalent cancer, with 2.26 million new cases and 680,000 deaths annually [1]. In the US, breast cancer represents 31% of all female malignancies [2], with triple-negative breast cancer (TNBC) accounting for 15–20% of cases; TNBC is aggressive and has a poor prognosis [3,4]. High-income countries bear a significant economic burden from breast cancer [5,6].

Molecular studies indicate that TNBC patients present an elevated TMB, increased PD-L1 expression, and increased presence of TILs [7–9], making TNBC the most immunogenic breast cancer variant and a prime candidate for immunotherapy benefits [10, 11]. For example, the combination of atezolizumab and nab-paclitaxel provides an effective immunotherapy option for US TNBC patients, but its cost-effectiveness is not significant compared with that of nab-paclitaxel alone [12]. Studies have also shown that the average monthly cost of first-line treatment for patients with metastatic TNBC in the United States is as high as $23,708.99, and this high cost highlights the importance of selecting more cost-effective first-line treatment options when treating patients with advanced TNBC in the United States [13].

As clinical data on immunotherapies accumulate, significant strides have been made in TNBC treatment [3,14,15]. Toripalimab, an inaugural PD-1 inhibitor from China, selectively targets PD-1 to unleash the body’s immune response against cancer [16, 17]. FDA approval for toripalimab in nasopharyngeal carcinoma marks it as the first Chinese antibody drug in the US market, with indications for both monotherapy and combination chemotherapy [18–20]. Pending FDA approval for use with nab-paclitaxel in the treatment of PD-L1-positive, untreated metastatic or recurrent TNBC, toripalimab could revolutionize US TNBC immunotherapy, offering new hope for patients [20–22].

The TORCHLIGHT trial, the only phase III study with affirmative outcomes in first-line treatment of TNBC, heralds a new age of immunotherapy for advanced TNBC. The interim analysis revealed that the combination of toripalimab and chemotherapy notably increased progression-free survival (PFS) in PD-L1-positive patients, decreasing the risk of progression or death by 35%, with a comparable PFS trend in the intention-to-treat (ITT) group. A significant improvement in overall survival (OS) was noted across all patients receiving toripalimab plus chemotherapy, with median OS times of 32.80 versus 19.50 months for PD-L1-positive patients and 33.10 versus 23.50 months for ITT patients. This treatment was well-tolerated and presented no novel safety concerns [23].

The outcomes of the TORCHLIGHT study signal a potential breakthrough, positioning toripalimab as a pioneering immunotherapy for first-line advanced TNBC treatment in the US, with the potential to fill existing treatment gaps [24–26]. However, toripalimab’s high cost raises concerns about economic strain and the need for improved cost-effectiveness to ensure drug accessibility. Our analysis aimed to assess the economic viability of incorporating toripalimab into first-line chemotherapy for advanced TNBC in the US health care framework.

## Materials and methods

### Model overview

**Fig. 1:**
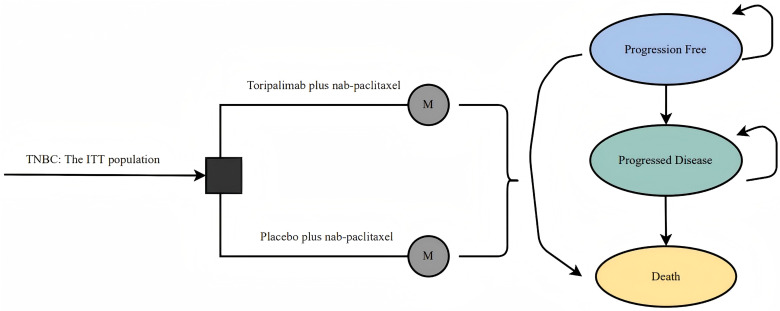
Decision tree and model structure overview. ***TNBC*** triple-negative breast cancer; ***ITT*** intention-to-treat; ***M*** Markov model.

A Markov model was applied to assess the cost-effectiveness of initial treatments for advanced TNBC in the US, considering the ITT population. The model included three states, PFS, PD, and death, mirroring the patient demographics of the TORCHLIGHT study. The treatments used were as follows: (1) toripalimab with chemotherapy, involving toripalimab 240 mg and nab-paclitaxel on specified cycle days; and (2) chemotherapy alone, with 125mg/m^2^ nab-paclitaxel on set days. Both treatments, capped at 2 years, continued until disease progression or intolerable side effects occurred. Post-progression, patients in both groups could transition to subsequent therapies until death (Fig 1). On the basis of TORCHLIGHT, it was assumed that patients in both treatment arms would receive up to 35 weeks of subsequent therapy following disease progression. This cost-effectiveness analysis was based on a literature review and modeling techniques. The study did not require approval from an institutional research ethics board.

The initial age of the model cohort was 53 years, and the Markov cycle length was 21 days. These values are consistent with the treatment cycles reported in the TORCHLIGHT study, with the model set at 5 years, a time frame that not only covers the critical survival of patients with advanced TNBC. The time frame also coincides with 5-year survival, an important clinical prognostic indicator, considering the time frame commonly used in pharmacoeconomic evaluations to balance short- and long-term cost-effectiveness and reduce uncertainty in model prediction. The primary endpoints of the model included overall mean costs, life years (LYs), quality-adjusted life years (QALYs), and incremental cost‒effectiveness ratios [27]. In this study, from the perspective of US payers, 100,000/QALY was used as a willingness-to-pay (WTP) threshold, which is consistent with US payers’ willingness to pay for new drugs and is consistent with local practice and policy frameworks [28, 29]. We estimated the ICER, i.e., the cost per additional QALY, by calculating costs and utilities at an annual discount rate of 5% per year [28] and compared it with the WTP threshold to assess the cost-effectiveness of atezolizumab combined with chemotherapy versus nab-P chemotherapy alone in the treatment of advanced TNBC [29]. All patients included in the model were disease-free. TreeAge Pro software was used in this study (https://www.treeage.com/) to construct and analyze the model.

### Model transition probabilities and survival estimates

Using the TORCHLIGHT study’s reported clinical data on efficacy and safety, we projected progression-free survival (PFS) and overall survival (OS) curves following Guyot et al.’s statistical methodology [30]. Given the nonparallel nature of these curves, we reject the proportional hazards (PH) assumption and instead apply an accelerated failure time (AFT) model. This model was effectively calibrated to the reconstructed individual patient data (IPD) using Stata 16 software.

Initially, data points were extracted from the PFS and OS curves using GetData Graph Digitizer software (version 2.26; available at http://www.getdata-graphdigitizer.com/index.php) and individual patient data were reconstructed using R software. The parametric survival functions were subsequently fitted to the PFS and OS curves in STATA 16. Here, model fit was evaluated using the Akaike information criterion (AIC), the values of which are presented in Table 1. Table 2 details the treatment models and estimated survival parameters for both toripalimab plus chemotherapy and chemotherapy alone, with fitted curves aligning closely to the Kaplan‒Meier curves from the TORCHLIGHT trial. Ultimately, cycle-specific transition probabilities were derived from survival parameters and functions for each PFS and OS curve.

**Table 1:** Summary of the goodness of statistical fit of the KM curve in the TORCHLIGHT trial.

	Exponential	Weibull	Gompertz	Lognormal	Loglogistic
Toripalimab plus nab-paclitaxel PFS curve for patients with metastatic or recurrent triple-negative breast cancer.					
AIC	765.7999	763.2804	766.9381	729.6481	738.8032
Toripalimab plus nab-paclitaxel OS curve for patients with metastatic or recurrent triple-negative breast cancer.					
AIC	570.5682	555.0409	566.4017	545.1305	549.7317
Placebo plus nab-paclitaxel PFS curves for patients with metastatic or recurrent triple-negative breast cancer.					
AIC	394.8166	388.0251	396.7134	365.9166	369.2248
Plus nab-paclitaxel OS curves for patients with metastatic or recurrent triple-negative breast cancer.					
AIC	336.5369	321.8711	330.9759	317.7559	317.8803

**Table 2:** Model parameters: baseline values, ranges, and distributions for sensitivity analysis.

Parameter	Value	Range	Distribution	Ref
Survival				
Toripalimab plus nab-paclitaxel group				
Loglogistic PFS curve of metastatic or recurrent triple-negative breast cancer.	λ = 0.11338; γ = 0.64072	–	–	[31]
Loglogistic OS curve of metastatic or recurrent triple-negative breast cancer.	λ = 0.03223; γ = 0.60082	–	–	[31]
Placebo plus nab-paclitaxel group				
Loglogistic PFS c urve of metastatic or recurrent triple-negative breast cancer.	λ = 0.14152; γ = 0.53806;	–	–	[31]
Loglogistic OS curve of metastatic or recurrent triple-negative breast cancer.	λ = 0.04349; γ = 0.53309	–	–	[31]
Costs $				
Drug cost, US $/per cycle				
Toripalimab (240 mg)	8,892.03	7,113.62 -10,670.44	Gamma	Yaozh.
Nab-Paclitaxel (100 mg)	1,447.14	1,157.71- 1,736.57	Gamma	[12]
Subsequent therapy	6,533.66	5,226.93- 7840.40	Gamma	[12]
Laboratory testing	16.36	13.09- 19.63	Gamma	[12]
CT scan (chest/abdominal/pelvis)	152.62	122.09- 183.14	Gamma	[12]
Tumor imaging	105	84 -126	Gamma	[32]
Terminal care per patient	85,904	68, 723-103, 085	Gamma	[32]
Carboplatin (1 mg)	0.06	0.04 – 0.07	Gamma	[33]
Gemcitabine	0.02	0.02 – 0.03	Gamma	[33]
Body surface area (m2)	1.84	1.78 – 1.90	Normal	[33]
Cost Administration, US$				
Cost AEs, US$				
Leukopenia	57.21	45.76-68.65	Gamma	[34]
Neutropenia	24,376	12,188-49,913	Gamma	[33]
Utilities				
Toripalimab plus nab-paclitaxel group				
PFS state	0.76	0.61-0.91	Beta	[12]
PD state	0.55	0.36-0.55	Beta	[12]
Leukopenia	0.09	0.07-0.10	Beta	[32]
Neutropenia	0.10	0.09-0.11	Beta	[12]
Risk for treatment-related AEs				
Toripalimab plus nab-paclitaxel group				
Leukopenia	0.25	0.20-0.30	Beta	[31]
Neutropenia	0.26	0.21-0.31	Beta	[31]
Placebo plus nab-paclitaxel group				
Leukopenia	0.23	0.18-0.28	Beta	[31]
Neutropenia	0.28	0.22-0.34	Beta	[31]

### Utility estimate

Patient health utilities for the two different treatment regimens were derived from published literature that quantified patient preferences for different health conditions [12,32], with utility values ranging from 0 (death) to 1 (full health) and negative values indicating worse disease states than death. We took advantage of these utility parameters to integrate them into the model for patients with advanced TNBC. The model assigns the corresponding utility values according to the health status of the patient to estimate QALYs, and the specific utility parameters are shown in Table 2. Moreover, we evaluated the uncertainty of the utility values in a sensitivity analysis.

### Cost estimate

The model focuses on direct medical expenses related to breast cancer treatment, including drug costs, progression management, routine follow-up, and severe adverse event (SAE, Grade ≥ 3) management costs, as well as hospice care expenses [12,32–34]. In this study, we included only the costs of grade ≥ 3 adverse events, mainly because these events have a significant impact on patients’ health and health care resource consumption [29]. The cost of toripalimab was sourced from Junshi Biological’s US partner, Coherus BioSciences, whereas subsequent treatment costs were based on published data. For model simplicity, we included only SAEs with an incidence of ≥ 5% for both treatments, assumed that all associated costs occurred in the initial cycle, and evaluated these assumptions in sensitivity analyses. The incidence of adverse events (AEs) was analyzed using data from the TORCHLIGHT trial, with management costs and other health care expenses detailed in Table 2, which were derived from the literature [33, 34].

### Sensitivity analysis

To assess the robustness of our model, we employed both univariate and probabilistic sensitivity analyses (PSAs). In the univariate analysis, we varied each parameter by ±  25% around its baseline value, as documented in Table 2, to identify which parameters significantly influenced the outcomes, as visualized through tornado diagrams. For PSA, we conducted 100,000 Monte Carlo simulations to determine the cost-effectiveness probability of toripalimab combined with chemotherapy. Each parameter in the model was assigned a specific distribution type, and values were randomly sampled within the defined ranges (as detailed in Table 2) for these simulations.

## Results

### Baseline results

From the viewpoint of the US. Compared with chemotherapy alone, the model’s 5-year projection indicates that incorporating toripalimab into first-line chemotherapy for advanced TNBC patients would result in an extra 0.41 LYs and an additional 0.29 quality-adjusted life years (QALYs), with increased costs amounting to $177,295.52. The analysis revealed that the ICER for toripalimab plus chemotherapy relative to chemotherapy alone was $593,750.61 per QALY, as detailed in Table 3. Although the ICER value of this study was higher than that of atezolizumab combined with nab-paclitaxel in patients with advanced TNBC in the United States ($242, 461.27/QALY) [12], this may be due to the initial high cost of new treatments. The cost of combining toripalimab with chemotherapy is expected to decrease over time, potentially improving future ICER values. In addition, this new regimen provides more treatment options for TNBC patients, even if at a higher cost, and may be of special value because of its pertinence.

**Table 3:** Base-case results.

Strategies and Scenarios	Total cost, $	LYs	QALYs	ICER ($/QALY)
Metastatic or recurrent triple-negative breast cancer.				
Toripalimab plus nab-paclitaxel	492,061.52	2.68	1.72	593, 750.61
Plus nab-paclitaxel Placebo	314,766.00	2.20	1.42	–

Table 3 outlines the model’s findings. Specifically, the cost per QALY for continuing therapies after post-disease progression until death is $492,061.52 greater for toripalimab combined with chemotherapy than for chemotherapy alone for US patients with advanced TNBC, indicating greater costs with similar therapeutic effects for the combination regimen.

### Sensitivity analysis results

**Fig. 2:**
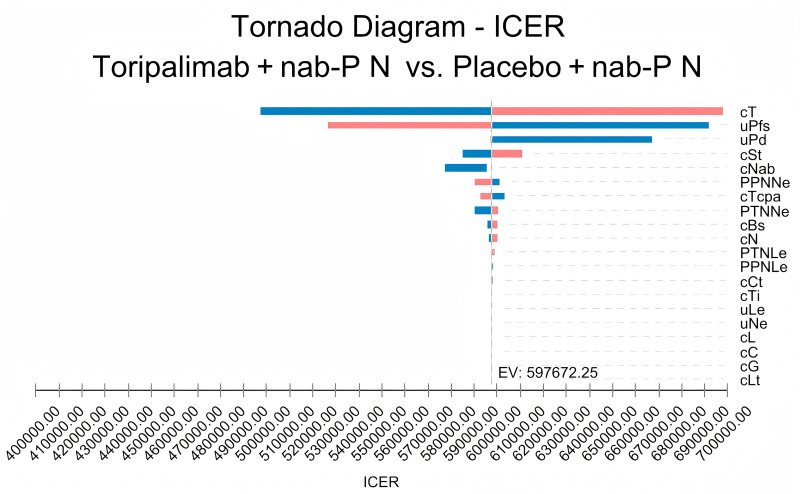
One-way sensitivity analysis tornado diagram of the incremental cost–effectiveness ratio (cost per quality-adjusted life-year) of toripalimab plus chemotherapy versus chemotherapy. cT, cost per cycle of toripalimab; uPfs, utility of subsequent free survival cycle; uPD, utility of progressive disease; cSt, cost of subsequent treatment per cycle after disease progression; cNab, cost per cycle of nab-paclitaxel; PPNe, probability of care in the PN group; cTcpa, utility of neutropenia per cycle; PTNNe, probability of care in the T+  N group; experiencing terminal cost cBs, carboplatin body surface area (m^2^); cN, cost per cycle of neutropenia; PTNLe, probability of experiencing leukopenia in the T +  N group; PPNLe, probability of experiencing leukopenia in the PN group; cCt, cost of CT scan; cTi, cost of tumor imaging; ucost, cost of leukopenia; uNe, cost of leukopenia; cL, cost of leukopenia; cC, cost of utility (1 mg); cG, cost of gemcitabine; cLt, cost of laboratory testing.

**Fig. 3:**
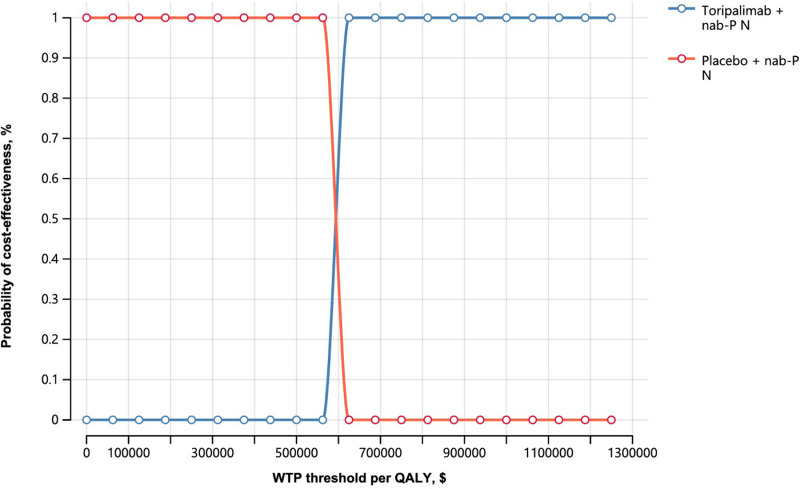
Cost-effectiveness acceptability curve for comparisons among various treatment regimens. CE, cost-effectiveness.

The univariate sensitivity analysis identified the cost of toripalimab per 240 mg dose and the utility values of progression-free (PFS) and progressive disease (PD) states as the most influential parameters affecting ICERs for advanced TNBC patients in the US, with other parameters having minimal impact, making the ICER relatively insensitive to adverse event treatment costs (Fig 2). The probabilistic sensitivity analysis, which was conducted with varied model inputs, resulted in a cost-effectiveness acceptance curve (Fig 3) indicating a 100% probability of cost-effectiveness for toripalimab plus chemotherapy versus chemotherapy alone at a $100,000/QALY willingness-to-pay (WTP) threshold for the US. advanced TNBC population in the US.

## Discussion

This analysis highlights the inaugural evaluation of the cost-effectiveness of toripalimab combined with chemotherapy versus chemotherapy alone for first-line treatment of advanced TNBC in the US from a payer’s standpoint. The TORCHLIGHT trial indicated that toripalimab, as the first PD-1 inhibitor demonstrating a notable overall survival (OS) benefit when paired with chemotherapy for advanced TNBC, holds promise for extending patient survival [28]. However, our findings suggest that incorporating toripalimab into first-line therapy for advanced TNBC yields a high ICER, exceeding the $100,000/QALY willingness-to-pay threshold, thus potentially deeming it less cost-effective in the US. context. The stability of these results was confirmed using both univariate and probabilistic sensitivity analyses.

The TORCHLIGHT study represents a significant milestone as the initial breakthrough in immunotherapy for advanced TNBC. To date, there has been no reported health economic evaluation of toripalimab in combination with chemotherapy versus chemotherapy alone for first-line treatment of advanced TNBC in the US. The anticipation is for toripalimab to emerge as a pioneering and exclusive immune checkpoint inhibitor sanctioned for first-line advanced TNBC treatment in the US., potentially increasing survival outcomes for patients with this condition.

Xiuhua Weng conducted a cost-effectiveness analysis of atezolizumab plus nab-paclitaxel versus nab-paclitaxel alone for first-line treatment of advanced TNBC in various countries on the basis of the IMpassion130 trial. For the US ITT population, the ICER for atezolizumab plus nab-paclitaxel compared with nab-paclitaxel alone was $242,461.27 per QALY and $331,996.89 per QALY [12]. Univariate sensitivity analysis in Weng’s study, aligning with our findings, highlighted the cost of PD-L1 or PD-1 inhibitors as the primary determinant of model outcomes. Weng’s study revealed that atezolizumab plus nab-paclitaxel was 0% cost-effective for advanced TNBC in the US, a result mirrored by our study’s assessment of toripalimab plus chemotherapy. These results suggest that both studies, sharing the same US advanced TNBC patient population and health utility values (PFS: 0.76, PD: 0.55), are comparable.

In the context of postprogression treatment, aligning with the TORCHLIGHT trial’s assumption that US patients with TNBC receive uniform subsequent therapies after disease progression, our cost-effectiveness analysis yielded conclusions that were consistent with those of the base-case analysis. This consistency, attributed to the similar therapeutic strategies for identical patient cohorts, offers valuable insights for policy-makers in the judicious allocation of health care resources.

Our analysis acknowledges several limitations. First, the Kaplan‒Meier survival curves from the TORCHLIGHT trial were utilized to project long-term drug effects, extending beyond the actual trial follow-up period. This may introduce discrepancies between model outcomes and real-world scenarios. To mitigate the impact of extrapolation on the results, we employed parametric models for long-term survival prediction and assessed the impact of extrapolation assumptions on the ICER using sensitivity analysis. Moreover, discount rates were applied rationally, and the transparency of the model assumptions was maintained to enhance the robustness and credibility of the findings. These measures help explain potential deviations between the model results and the actual situation. Second, key clinical costs, such as subsequent treatment costs for PD, were sourced from the literature rather than from study-specific surveys and included only expenses for Grade III/IV adverse events affecting ≥ 5% of the TORCHLIGHT participants, potentially skewing the estimation of the cost of AEs. Sensitivity analyses indicated that ICERs were relatively insensitive to these AE costs. Third, there is uncertainty regarding long-term survival predictions from the TORCHLIGHT trial, without subgroup analyses, necessitating ongoing data updates to affirm our model’s results. Nonetheless, we posit that our study provides a reflection of clinical treatment of US TNBC patients using toripalimab in combination with nab-paclitaxel as a first-line treatment.

In summary, although options for immunotherapy combined with chemotherapy for TNBC in the US are limited, the TORCHLIGHT trial’s ITT population demonstrated benefits from toripalimab plus chemotherapy, suggesting that it is a viable treatment option for advanced TNBC patients in the US. when feasible. Our study pioneers the cost-effectiveness evaluation of this combination as a first-line therapy, highlighting the substantial improvement in OS attributed to toripalimab. The analysis also revealed that histology has a minimal influence on the cost-effectiveness of toripalimab in this context. For advanced TNBC patients in the US who have not undergone PD-1 testing, initiating treatment with toripalimab plus chemotherapy may not be cost- effective. Implementing a price negotiation or alternative payment model for toripalimab may be one of the most effective measures to improve the cost-effectiveness of first-line toripalimab in combination with chemotherapy strategies for patients with advanced TNBC in the United States.

In this study, we evaluated the cost-effectiveness ratio of toripalimab combined with nab-paclitaxel as a first-line treatment for advanced TNBC in the United States using cost-effectiveness analysis, providing evidence-based decision support for health policy-makers to assist in resource optimization allocation. The results provide a scientific basis for pricing, purchasing, and reimbursement decisions for toripalimab, while helping to control health care costs and improve health system efficiency. In addition, the findings directly impact market access and price negotiations for toripalimab highlighting the importance of cost-effectiveness advantages. These findings not only deepen the understanding of the cost-effectiveness of this combination regimen, but also provide a reference for future research and policy development.

## Conclusion

From the perspective of the US health care system, toripalimab combined with chemotherapy lacks a cost-effective advantage over nab-P chemotherapy as a first-line treatment of patients with advanced TNBC in the United States at a WTP threshold of $100,000/QALY. Therefore, further exploration of price negotiations or alternative payment models for toripalimab may be an effective method to increase the cost-effectiveness of this combination chemotherapy strategy, thereby optimizing its accessibility in clinical practice.

## Abbreviations

TNBC: Triple-negative breast cancer
ICER: Incremental cost-effectiveness ratio
LY: Life year
QALY: Quality-adjusted life year
WTP: Willingness to pay
EGFR: Epidermal growth factor receptor
AEs: Adverse events
PFS: Progression-free survival
GDP: Gross domestic product
IPD: Individual patient data
AIC: Akaike information criterion
PSA: Probabilistic sensitivity analysis
